# Persistent Vitality

**Published:** 1894-08

**Authors:** F. O. Jacobs


### Persistent Vitality.

BY F. O. JACOBS, D.D.S.

Read before the Meeting of the California State Dental Association, 1894.

The interest that has lately been created in the operations of replantation, transplantation, etc., has raised the question among dentists as to whether a tissue will survive after having been removed from its natural position and again replaced, or after having been removed, can it again be transplanted to another individual without suffering death? And if so what time may elapse between the two operations? And if it should be determined that such operations were possible what tissues are the most favorable to such operations? Or, in other words, and more briefly, is there such a thing as persistent vitality?

In surgical operations, just as in every other department of science, we are constantly meeting with surprises. What had been classed with the impossible yesterday is the practicable operation of to-day, and we are constantly being driven back to first principles to account for some unexpected results.

This subject involved the broad question, what are the conditions that produce death, and what conditions are required to support life? Can we lay down any law or rules that will hold good in all cases? At first we meet with so many seeming contradictions that we are discouraged in the attempt. We are prone to think that one form of life is something entirely different from another, and the rules that would apply to one will not apply to another. While we find, in general, that living organisms may exist only in an atmosphere or medium containing more or less free oxygen, some one comes along and tells you he knows of certain forms of life that can exist in an atmosphere of carbonic acid, drawing all its oxygen from its food. Another must have a medium of salt water; another must exist in fresh water; others still cannot be immersed in water but for a few moments without suffering death. Some organisms are able to withstand extreme changes of moisture or temperature. Some animal forms and also plants may be deprived entirely of their moisture—completely dried up, so that vital functions are entirely suspended until moisture is again restored. Some may be frozen in cakes of ice or raised to a temperature near the boiling point of water without any other effect than to suspend functional activity for the time being.

Among the more highly-specialized organisms the amputation of a member or organ generally produces death in the parts so treated, or in the entire organism, owing to which organ or part is separated from the body. But in the more simple forms amputation of a part only produces a new animal. A remarkable example of this is found in the fresh water hydra. This animal consists of a tube, one end of which is modified into a foot and the other is furnished with arms. The entire animal is of but one tissue. If an arm is amputated it immediately grows into a new hydra. The animal may be cut into several pieces, with the result of producing as many animals instead of death. One might think it was possessed of remarkable tenacity of life, but these animals are as easily killed as any. Turn the animal inside out, it immediately makes an effort to right itself. Should it fail, it dies. Deprive it of moisture and it dies.

Why should the cutting of an animal to pieces only have the effect of multiplication, and simply depriving it of moisture produce death? Exactly the reverse of some other plants or animals. The explanation is probably this: its structure is that of large, delicate cells, very soft, which are easily broken up by desiccation, so that by depriving it of moisture its structure is destroyed; death is, of course, the result. Cutting it in pieces only multiplies because it is composed of but one tissue throughout, capable of performing the same functions. Hence, a piece, if separated from the balance of the animal, is not dependent upon it to perform any of the functions necessary to support life. Reversing destroys its life, because the cells on the inside of the tube are so modified as to perform the functions of assimilating food; while the outer cells are for the protection of the inner. When the animal is reversed neither of these sets of cells are in a position to perform their natural functions, therefore, death is the result.

Now, there are other plants and animals of which the reverse is true, which if deprived of their moisture simply remain inactive until the proper quantity of moisture is restored when they resume their normal functions. In their case the cell structure is a strong, firm tissue, which is not broken up by desiccation. Depriving the living matter or protoplasm within of its moisture is not a chemical but a physical change. Chemical action, oxidation, etc., goes on extremely slow, if at all, until the proper conditions are again restored for the performance of its natural functions. The living matter within the cells is a chemical compound, the exact chemical nature of which is not known, but its constituent elements are known. It is extremely unstable, being constantly acted upon by free oxygen on the one hand, it must be constantly taking up the proper material on the other to preserve the equilibrium in its chemical nature. Its affinity for water is great and its activity depends upon the presence of water; the more water it is able to absorb, the more active it becomes. In the absence of that element activity ceases until moisture is restored, unless some chemical change takes place, when it is no longer protoplasm. As it does not form compounds with other substances, without losing its identity, differing in the least from itself in chemical nature, a living tissue is never attached to a dead one or to other substances. Hence, when a living tissue is found attached to another, supposed to be a dead one, it must be considered evidence that the tissue supposed to be dead is at least possessed of a low degree of vitality.

What has been said of protoplasm is true of it wherever found, for living matter is the same thing wherever found. The different modes of death cited above are due to the difference in the structure of the tissue it inhabits. Where the tissue is composed of firm, hard cell-walls it is much more able to endure desiccation. Where an organism is composed of a similar tissue throughout its entire body, separating it into pieces only multiplies it.

One of the present methods of skin-grafting, scraping the epithelial cells from the surface of the skin on to the part to be engrafted, is a case where a tissue, or cells of formed tissue, is engrafted on to another tissue, forming a union and a new tissue; transplanting.

Now, if a tissue can be transplanted from one place or location to another, some time must elapse between the two operations. It then becomes a question how long will a tissue endure between the two operations, and what tissues are most favorable to such operations? How long could the scrapings of epithelial cells be preserved before transplanting, or how much time might elapse between the operation of extracting and of transplanting a tooth? The evidence furnished by such operations is conclusive, that it is much greater than any of us have supposed. Bone may be grafted from one animal to another, even chips of bones may be placed into the wound and a new tissue formed around it. (American Text-Book of Surgery.)

Why may not a tooth be successfully transplanted? Its structure is not changed by extracting, and decomposition is less likely to set in than in any other tissue of the body.

The above illustration may seem to be far-fetched, but not so. Much of what we have yet to learn in physiology must be drawn from the study of minute and simple formed organisms. The facts to which I have called your attention will show that death implies a chemical change, and without that there is no death.

It also shows that the tissues that are best suited to transplanting, replanting, etc., are hard, firm and simple structured tissues; like the skin, the teeth and bones. Those less suited for such operations are of delicate cell-walls and soft, loose structure, that will easily collapse when removed from its normal position, and by loss of moisture.

The success that has been met with in transplanting such tissues fully confirms the above conclusion.

